# Hydration-Mediated Multiscale Structural Evolution in Polyurethane Hydrogels: From Water States to Microphase Reconstruction and Dynamic Networks

**DOI:** 10.3390/gels12100911

**Published:** 2026-10-10

**Authors:** Li Zhang, Rongrong Zhang, Xinliang Wang, Honghong Zhang, Zhaosheng Hou

**Affiliations:** 1 College of Chemical Engineering and Materials, Shandong University of Aeronautics, Binzhou 256600, China; zhanghong@sdua.edu.cn; 2 Shandong Key Laboratory of Chemical Process Simulation and Optimization Industrial Software (PKL2024F23), Qingdao 266042, China; 3 College of Chemistry, Chemical Engineering and Materials Science, Shandong Normal University, Jinan 250014, China; 2025214102l@stu.sdnu.edu.cn; 4 Beijing WholesomeTech Co., Ltd., Beijing 101500, China; 18053100788@163.com

**Keywords:** polyurethane hydrogels, hydration, water state, hydrogen bonding, microphase separation, microphase reconstruction, dynamic networks, wet-state mechanics

## Abstract

Polyurethane (PU) hydrogels combine high water content with the intrinsic structural heterogeneity of segmented polyurethane, giving rise to strong coupling among hydration, intermolecular interactions, microphase organization, and network dynamics. This review treats hydration as an active structural variable rather than solely as a swelling medium. The effects of soft- and hard-segment chemistry and network topology on the pre-hydration state are first summarized, followed by analysis of the multiscale sequence from water uptake and water state to competitive hydrogen bonding, segmental mobility, microphase reconstruction, and dynamic-network evolution. Particular attention is paid to distinguishing hydration-induced reconstruction of pre-existing domains from water-induced microphase separation, in which hydration generates new compositional segregation or phase connectivity. Direct evidence from PU hydrogels is also distinguished from mechanistic evidence derived from related hydrated PU, polyurethane–urea, and thermoplastic PU systems. The consequences of these processes for strengthening, toughening, cyclic recovery, stimuli responsiveness, wet interfaces, and representative applications are discussed. A system-dependent hydration window is further introduced to define the conditions under which sufficient molecular mobility for reversible reorganization coexists with persistent load-bearing structures. Remaining challenges include synchronized wet-state characterization, long-term fatigue and aging, and the establishment of predictive composition–hydration–structure–property relationships.

## 1. Introduction

### 1.1. Hydrogel Development and Structure–Property Trade-Offs

Hydrogels are water-rich polymer networks whose softness, permeability, and mass-transport capability have enabled broad applications in tissue engineering, drug delivery, flexible devices, and soft actuation [1,2,3,4,5]. As these applications increasingly require simultaneous load bearing, large deformation, adhesion, and environmental responsiveness, hydrogel design has evolved from simply controlling swelling or crosslink density to constructing networks that combine stable load-bearing structures with dissipative and reconstructable motifs. Double-network, ionic-covalent, and polyampholyte hydrogels have demonstrated that permanent networks, sacrificial structures, and reversible interactions can be combined to improve mechanical strength and energy dissipation while retaining high water contents [6,7,8].

These advances also highlight intrinsic trade-offs in hydrated polymer networks. Increasing permanent crosslink density generally improves stiffness and dimensional stability but restricts segmental mobility, whereas reversible interactions enhance energy dissipation and structural rearrangement but may also lead to greater hysteresis or slower recovery [8,9,10]. Moreover, toughness under a single loading event is distinct from resistance to cyclic crack growth; repeated dissociation of sacrificial structures can progressively accumulate damage when recovery is incomplete [11]. The central challenge is therefore not to maximize any single property, but to coordinate hydration, load-bearing stability, molecular mobility, energy dissipation, and structural recovery. Segmented polyurethane (PU), which combines chemically distinct soft and hard segments, abundant hydrogen-bonding sites, a tunable hydrophilic–hydrophobic balance, and intrinsic microphase organization, provides a particularly suitable platform for examining this competition between structural stability and dynamic reconstruction.

### 1.2. Structural Characteristics of Polyurethane Hydrogels

Unlike hydrogels based on relatively homogeneous hydrophilic networks, segmented PU contains chemically distinct soft and hard segments that generate intrinsic structural heterogeneity. Soft segments, typically derived from polyethers, polyesters, or polycarbonates, primarily regulate flexibility, segmental mobility, and water affinity, whereas urethane- and/or urea-rich hard segments can associate through hydrogen bonding and local ordering to form relatively cohesive domains. Differences in polarity, cohesive energy density, segmental regularity, and intermolecular interactions consequently give rise to varying degrees of microphase separation and phase mixing [12,13]. The resulting morphology depends on soft- and hard-segment chemistry, segment length, diisocyanate structure, hydrogen-bond organization, and composition [12,14].

This segmented architecture also generates heterogeneous hydration environments. Hydrophilic segments facilitate water uptake, whereas hydrophobic regions, hard-segment aggregates, crystallites, and network constraints can suppress excessive swelling [15,16]. Urethane and urea groups further participate in competing hard segment–hard segment and hard segment–soft segment hydrogen bonds, making microphase organization responsive to changes in local solvation and segmental mobility [12,14,17]. Consequently, water can act not only as a swelling medium but also as a structural perturbant or selective solvent. Depending on molecular composition and network constraints, hydration may weaken pre-existing physical associations, increase segmental mobility, or promote structural reorganization. The formation of bicontinuous PU hydrogel networks through in situ water-induced microphase separation provides a direct example of the latter behavior [18]. PU hydrogels are therefore more appropriately considered hydration-responsive heterogeneous networks than uniformly swollen hydrophilic matrices.

Current research on PU hydrogels can be broadly grouped into four overlapping design directions that address different limitations of conventional hydrated polymer networks. First, hydrophilic segmented PU hydrogels, particularly those containing poly(ethylene glycol)/poly(ethylene oxide) (PEG/PEO)-rich soft segments, remain important because they provide high water uptake, tissue-like compliance, and molecular transport while retaining network integrity through hard-segment association or additional crosslinking [12,15,16]. Second, microphase-engineered PU hydrogels deliberately exploit hydrophilic–hydrophobic heterogeneity and domain connectivity to reconcile high hydration with efficient load transfer and energy dissipation; water-induced bicontinuous PEG-PU/poly(ε-caprolactone)-based PU (PCL-PU) hydrogels represent a recent example in which hydration itself is used to construct a mechanically reinforcing morphology [18]. Third, dynamic and self-healing PU hydrogels incorporate reversible supramolecular or covalent junctions, including hydrogen bonds, disulfides, and acylhydrazones, to couple energy dissipation with network reconstruction and damage recovery [19]. Fourth, stimuli-responsive and injectable PU hydrogels use temperature-, pH-, redox-, or shear-dependent changes in polymer–water interactions and network connectivity to enable reversible swelling, sol–gel transitions, controlled release, or post-injection recovery [20,21,22]. These directions are increasingly combined rather than pursued independently, because advanced PU hydrogels are required to coordinate hydration, mechanical integrity, molecular mobility, structural recovery, and processability under their intended service conditions.

### 1.3. Limitations of Current Research and Scope of This Review

Previous literature has separately addressed segmented-PU structure–morphology–property relationships [12], PU microphase separation and its characterization [13], dynamic covalent PU networks and their self-healing and reprocessability [19], and biomedical/functional PU hydrogels [20,21,22]. Although comprehensive, these frameworks are organized mainly by composition, individual mechanisms, or applications rather than by hydration-mediated structural evolution. Hydration has also been extensively studied in the broader hydrogel literature. Gun’ko et al. summarized thermodynamically distinguishable water populations and their characterization by DSC, NMR, dielectric spectroscopy, and related methods [23], while recent reviews of phase-separated hydrogels linked polymer-rich/solvent-rich domain connectivity to toughness, fatigue resistance, adhesion, and stimuli responsiveness [24]. These concepts have rarely been integrated for segmented PU hydrogels. PU-specific studies show that water can alter segmental dynamics, hydrogen bonding, hard-domain stability, and stress relaxation [25], whereas selective hydration can induce in situ microphase separation and bicontinuous hydrogel structures [18]. The key question is therefore when hydration produces mainly plasticization and structural relaxation, and when it instead promotes microphase reconstruction and mechanical reinforcement.

**Figure 1:**
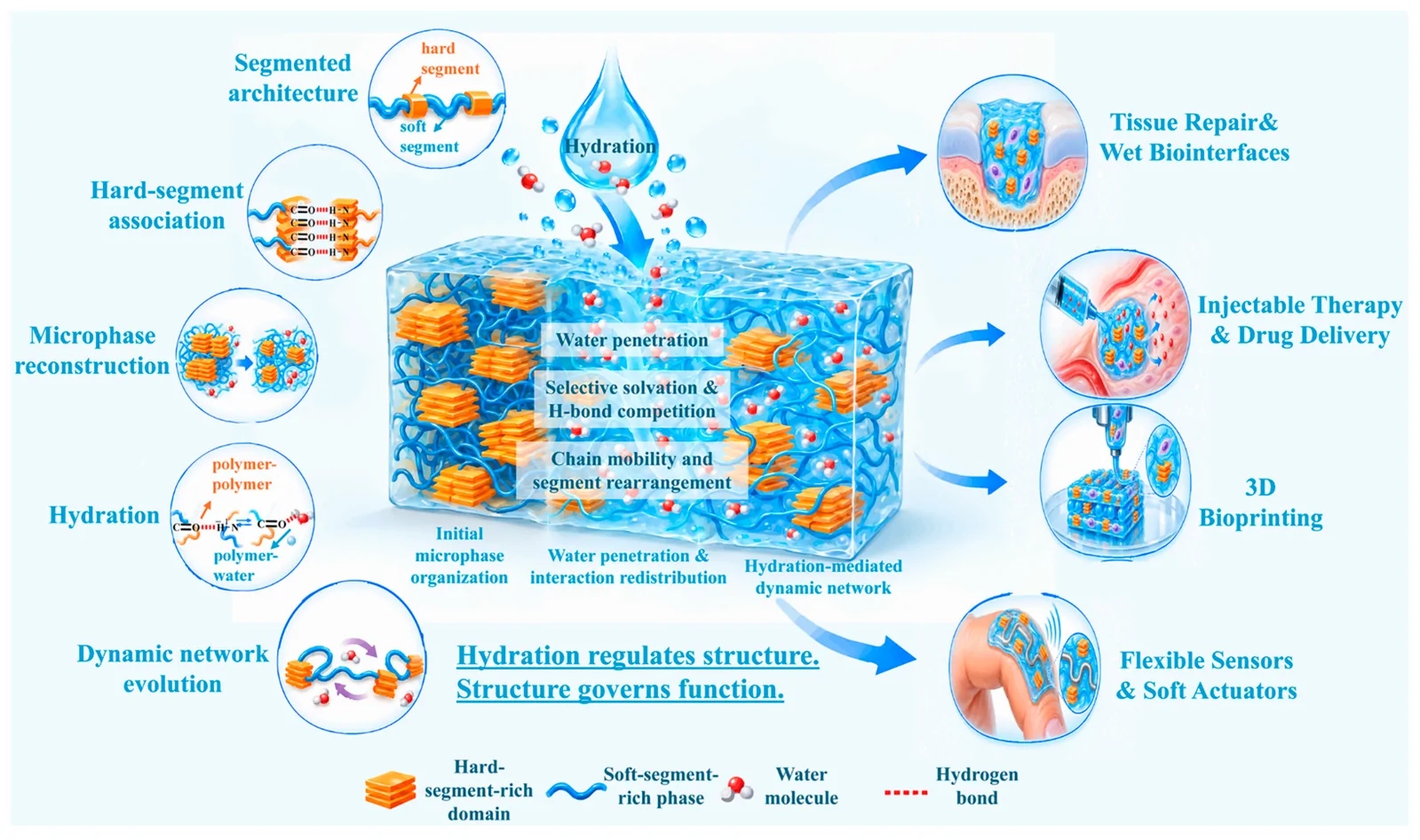
Conceptual illustration of hydration-mediated structural regulation in polyurethane hydrogels.

In this review, “high-water-content PU hydrogels” refers operationally to equilibrium-swollen, water-rich PU or polyurethane–urea (PUU) networks in which water constitutes a major component of the swollen state and directly influences molecular mobility, intermolecular interactions, transport, and/or macroscopic properties. The term is used as an operational description rather than a universal numerical cutoff because equilibrium water contents and experimentally defined water states vary among different systems and characterization methods [23]. Accordingly, this review focuses on water-rich systems in which PU or PUU forms the principal network or an important structural phase. Because direct in situ evidence for the full hydration–hydrogen-bonding–microphase sequence remains limited in equilibrium PU hydrogels, studies of hydrated PU, PUU, and thermoplastic polyurethane (TPU) are used as mechanistic evidence rather than equivalent quantitative validation. Hydration is treated as an active structural variable that links molecular and network architecture to water state and polymer–water interactions. These hydration-dependent interactions regulate competitive hydrogen bonding and segmental mobility, which in turn influence microphase reconstruction, dynamic-network evolution, and ultimately macroscopic properties and functions (Figure 1). Throughout, direct hydrogel evidence is distinguished from related hydrated-PU evidence, and unresolved cross-scale relationships are explicitly identified.

## 2. Molecular Architecture and Network Design of Polyurethane Hydrogels

The hydration response of a PU hydrogel is largely predetermined by the molecular and network structures established during synthesis. Soft- and hard-segment chemistry governs polarity, flexibility, intermolecular interactions, and thermodynamic compatibility, whereas hard-domain formation, crystallization, and crosslinking constrain segmental motion and volumetric swelling. Water uptake and hydration-induced reconstruction should therefore be interpreted as responses of a pre-existing heterogeneous network rather than as processes occurring in an initially structureless matrix. This section focuses on the molecular and network variables that define this pre-hydration structural state.

### 2.1. Segmented Architecture and the Pre-Hydration Structural State

**Figure 2:**
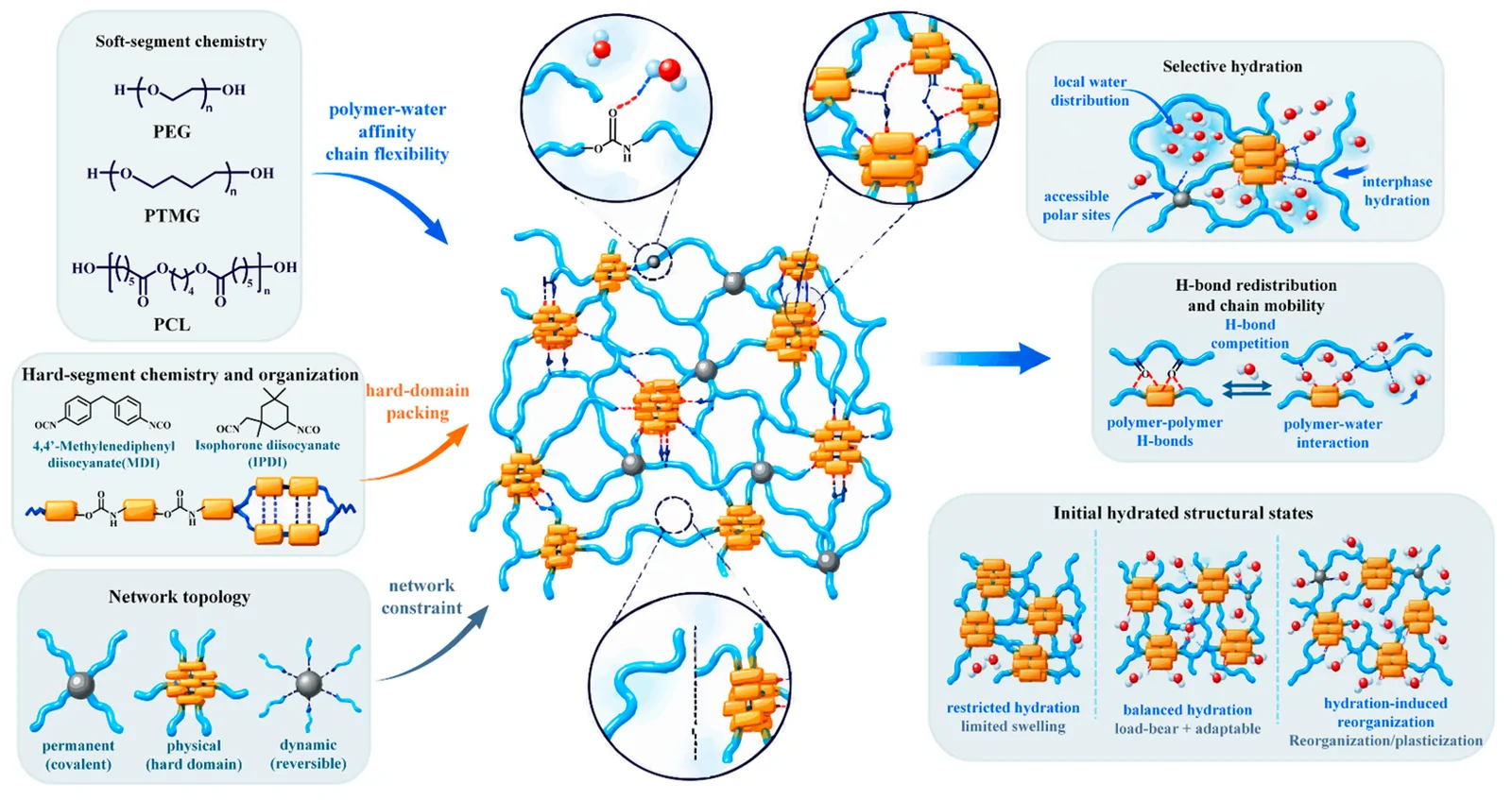
Representative molecular components and network determinants of hydration behavior in polyurethane hydrogels. Soft-segment chemistry, hard-segment chemistry and organization, and network topology collectively regulate polymer–water affinity, intermolecular interactions, segmental mobility, and the resulting hydrated structural states.

Segmented PU consists of chemically distinct flexible soft segments and more cohesive hard segments, and its initial morphology is governed by their chemical structures, molecular weights, relative contents, and compatibility. Yilgör et al. identified these variables, together with competing intermolecular interactions, as key determinants of microphase morphology and macroscopic properties [12]. In PU hydrogels, they additionally establish the spatial distribution and accessibility of hydrophilic regions, hydrophobic regions, and physically crosslinked domains before hydration. Soft-segment chemistry influences both water affinity and compatibility with hard segments. For example, variations among PEG-, poly(propylene glycol) (PPG)-, and ethylene oxide/propylene oxide (EO/PO)-containing segmented PUs produce different degrees of phase mixing and phase separation [26]. Hard-segment organization is likewise sensitive to molecular geometry and hydrogen-bonding capability; increased structural symmetry can facilitate more ordered packing and distinct microphase morphologies [14]. The respective contributions of soft-segment hydrophilicity and hard-segment association to hydration propensity are considered separately below. Representative soft-segment structures, hard-segment components, and major network-junction types are summarized schematically in Figure 2 to illustrate how molecular and network design regulates polymer–water affinity, intermolecular association, and the resulting hydrated structural state.

### 2.2. Soft-Segment Chemistry and Hydrophilic–Hydrophobic Balance

Soft segments primarily regulate chain mobility and polymer–water affinity. PEG/PEO provides abundant ether oxygens capable of hydrogen bonding with water and is therefore widely used to increase hydration of PU networks, whereas less hydrophilic polyethers, polyesters, polycarbonates, or hydrophobic structural units can reduce water affinity. Soft-segment chemistry and molecular weight also influence compatibility with hard segments and thus cannot be considered independently of microphase organization [12]. Experimental studies illustrate that hydration is controlled by a balance between water affinity and structural confinement rather than by hydrophilicity alone. Increasing PEG content in PEG/castor-oil-based PU hydrogels increased swelling and reduced the water contact angle [15]. In PEG–polyhedral oligomeric silsesquioxane (POSS) multiblock PU, PEG-rich segments promoted hydration, whereas hydrophobic POSS-rich nanodomains acted as physical crosslinks that preserved network integrity after swelling [16]. Accordingly, soft-segment chemistry should be tuned to provide sufficient water accessibility while avoiding excessive swelling that would compromise load-bearing connectivity. This balance defines the local molecular environment for subsequent polymer–water interactions and segmental rearrangement.

### 2.3. Hard-Segment Structure, Hydrogen Bonding, and Domain Formation

Hard segments constitute the principal high-cohesive-energy regions of segmented PU and can aggregate into physically crosslinked domains. Their organization depends on hard-segment content, diisocyanate structure, sequence regularity, molecular symmetry, and thermodynamic incompatibility with the soft phase [12,13]. Experimental and computational studies further show that molecular symmetry affects chain packing, hydrogen-bond organization, and the resulting degree of microphase separation [14]. Hydrogen bonding is a major molecular driving force underlying this organization. Urethane and urea N–H groups act as donors, whereas carbonyl groups and polar soft-segment functionalities can act as acceptors. Hard segment–hard segment hydrogen bonding generally favors aggregation and ordering, whereas hard segment–soft segment hydrogen bonding promotes interphase compatibility and phase mixing [12,14]. Urea-containing PUU systems can exhibit particularly strong and dense hydrogen-bond associations, leading to pronounced changes in interchain cohesion and phase organization [27,28].

Hard domains consequently function as physical crosslinks and load-transfer units, but they are not static. Continuous association and dissociation of hydrogen bonds have been demonstrated in segmented PU, indicating dynamic molecular exchange within hard-segment-rich regions [17]. The pre-hydration network therefore contains a hierarchy of relatively stable domain structures and reversible intermolecular interactions. How water perturbs this hierarchy is considered in Section 3.

### 2.4. Network Construction Strategies for Polyurethane Hydrogels

Molecular heterogeneity must ultimately be incorporated into a three-dimensional network that remains intact in water. According to the nature and lifetime of network junctions, PU hydrogels can be broadly described as permanent covalent, physical/supramolecular, dynamic covalent, or hybrid networks. These architectures regulate both structural stability and the degrees of freedom available for swelling and segmental rearrangement.

Permanent covalent crosslinks suppress chain dissolution and provide long-term dimensional stability, although increasing crosslink density generally limits swelling and segmental mobility. Triol-crosslinked degradable PU hydrogels demonstrate that covalent-network architecture can regulate wet-state mechanical and interfacial properties [29], while PEG content and crosslinking structure jointly influence water uptake and surface properties [15]. Physical or supramolecular networks instead rely on reversible junctions such as hydrogen bonding, hydrophobic association, hard-segment aggregation, and microphase separation. PEG–POSS multiblock PU provides an example in which phase-separated hydrophobic domains stabilize a hydrated network [16], whereas poly(vinyl alcohol) (PVA)-mediated physical crosslinking can regulate the structure and water uptake of waterborne PU hydrogels [30]. Dynamic covalent networks retain covalent connectivity while permitting topological rearrangement through reversible exchange reactions, including disulfide, imine, and acylhydrazone chemistry [31,32,33]. Compared with purely physical junctions, these bonds can provide greater network persistence while retaining time-dependent reconstructability. Recent PU hydrogels increasingly combine junctions with different strengths and exchange timescales. Dual-crosslinked networks can integrate permanent and reversible interactions within the same material [34], while tetra-PEG/disulfide-containing PU hydrogels illustrate the incorporation of reconstructable covalent motifs into stable networks [33]. Hydrogen bonding, coordination, and microphase-based physical crosslinking can further operate cooperatively with chemical networks [18,35]. The relevant design variable is therefore not crosslink density alone, but the hierarchy of junction strength, lifetime, spatial distribution, and mobility.

### 2.5. Integrated Determinants of Hydration Propensity

The hydration propensity of PU hydrogels is determined jointly by polymer–water affinity, polar-site accessibility, microphase organization, and network constraints rather than by hydrophilic-group content alone. Hydrophilic soft segments such as PEG/PEO generally favor water uptake, whereas hydrophobic regions, hard-segment aggregation, crystallization, and dense crosslinking restrict water penetration and volumetric expansion [12,15,16]. Networks with similar nominal hydrophilic contents can therefore exhibit different hydration behaviors because their polar groups reside in distinct local structural environments.

Importantly, these structural variables define only the initial propensity for hydration. Once water enters the network, it can alter segmental dynamics and pre-existing microphase organization [25] and, under appropriately designed hydrophilic–hydrophobic heterogeneity, can actively drive new structural organization [18]. Figure 2 summarizes this transition from molecular/network design to the initial hydrated state. The relevant question thus shifts from how much water is absorbed to where water resides, how strongly it interacts with the network, and how these interactions modify hydrogen bonding, segmental dynamics, and microphase structure.

## 3. Hydration-Mediated Multiscale Structural Evolution in Polyurethane Hydrogels

Hydration of segmented PU is spatially and temporally heterogeneous rather than a uniform expansion of the dry network. Water preferentially solvates accessible hydrophilic segments and polar sites, whereas hydrophobic regions and hard-segment-rich domains impose local constraints on penetration and rearrangement. Consequently, water uptake can alter polymer–polymer hydrogen bonding, segmental mobility, interfacial organization, and hard-domain stability over different timescales [25,36]. Understanding this process requires distinguishing total water uptake from water state and subsequently linking local solvation to molecular interactions, microphase evolution, and network dynamics. This section therefore examines how water state influences competitive hydrogen bonding and segmental mobility and how these molecular changes subsequently affect microphase reconstruction, together with the experimental evidence required to establish each step.

### 3.1. Water Uptake, Swelling, and Water State

Water uptake, swelling, and water state describe related but distinct aspects of hydration. Water uptake quantifies the amount of absorbed water, whereas swelling describes the associated mass or volume expansion. Water state instead reflects the local association and mobility of water within the polymer network. In heterogeneous PU hydrogels, similar equilibrium water contents may therefore correspond to different local hydration environments because soft-segment chemistry, polar-site accessibility, crosslinking constraints, and microphase organization influence where and how water is accommodated. On the basis of freezing and melting behavior, hydrogel water is commonly classified operationally as non-freezing water, freezable bound water, and free or bulk-like water. Non-freezing water interacts strongly with polymeric or ionic sites and does not exhibit a conventional bulk-water freezing transition; freezable bound water remains influenced by polymer chains or confinement but can crystallize at depressed temperatures; and free water exhibits behavior closer to that of bulk liquid water [23,37]. These categories are experimentally useful but should not be interpreted as three sharply separated spatial phases. Water in hydrogels instead spans a continuum of interaction strengths and molecular mobilities.

The term “bound water” is also method-dependent. DSC identifies water populations from freezing/melting transitions, whereas NMR and dielectric spectroscopy probe local chemical environments and molecular mobility over different timescales [38]. This distinction is particularly important for PU. In PU ionene hydrogels, DSC classified water associated with strongly interacting ionic sites as non-freezing water, whereas drying-based measurements additionally included water associated with PEG ether groups [39]. In other segmented PU systems, the absorbed water remained non-freezing within the detection capability of DSC at saturation [40], whereas highly swollen PU ionenes contained predominantly free or bulk-like water [39].

**Figure 3:**
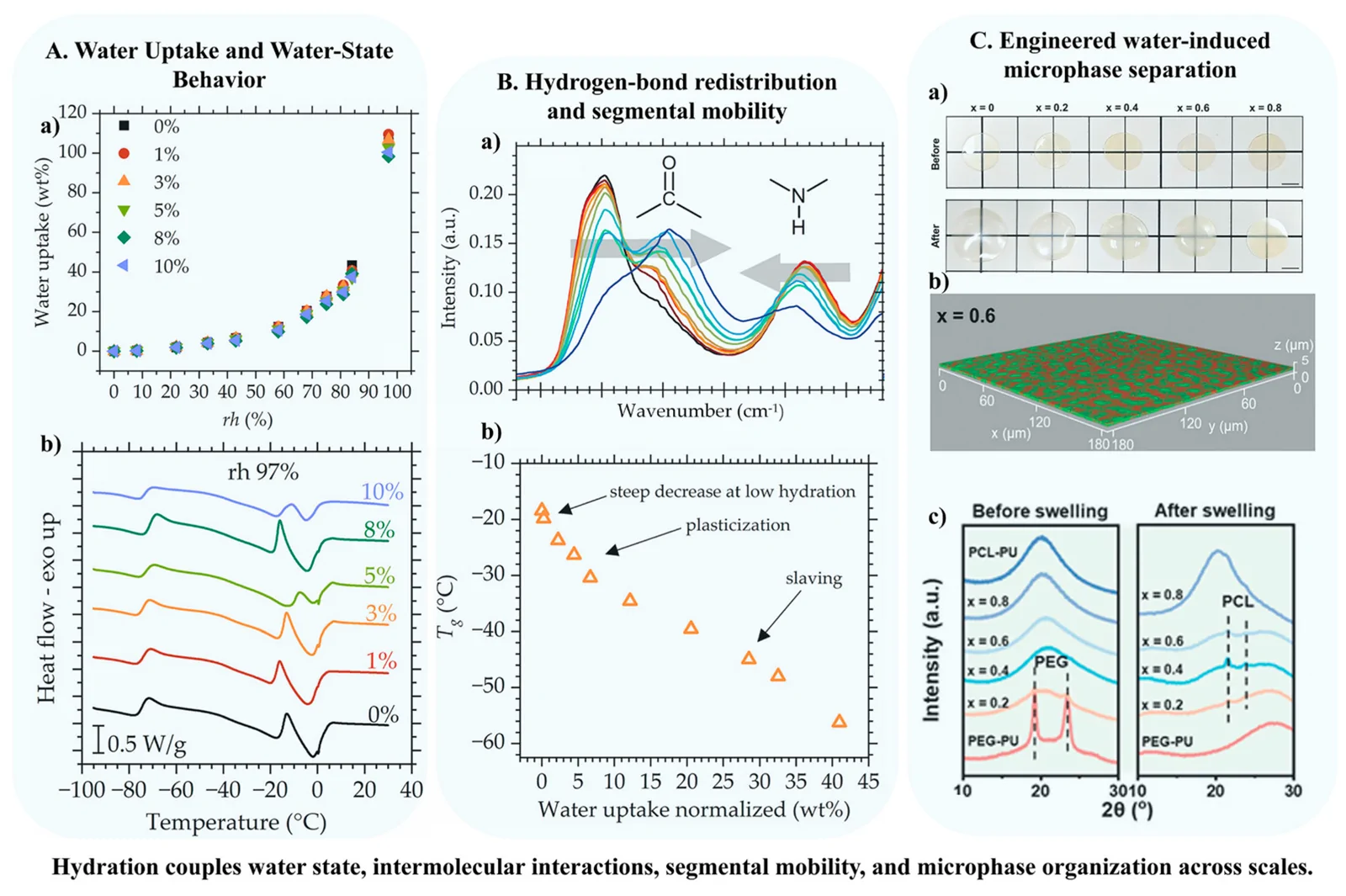
Representative multiscale experimental evidence for hydration-mediated structural evolution in polyurethane-based hydrogel systems. (**A**) Water uptake and water state in 8OHPOSS-modified NIPU networks: (**a**) equilibrium water-vapor sorption isotherms (0–10 wt% 8OHPOSS); (**b**) DSC thermograms at *rh* = 97%, showing cold crystallization and melting of freezable water. (**B**) Hydration-induced hydrogen-bond redistribution and segmental mobility: (**a**) FTIR spectra showing red-shifted C=O stretching and blue-shifted N–H bending bands, consistent with increased polymer–water hydrogen bonding; (**b**) *T*_g_ versus polymer-mass-normalized water uptake for 3 wt% POSS, showing plasticization at low-to-intermediate hydration and slaving at higher hydration. (**A**,**B**) Adapted from Ref. [41] under CC BY 4.0 (© 2023 by the authors). (**C**) Water-induced microphase separation in PEG-PU/PCL-PU hydrogels: (**a**) photographs before and after swelling at different PCL-PU mass fractions (x); (**b**) 3D confocal laser scanning microscopy (CLSM) reconstruction of PEG-PU/PCL-PU_0.6_ showing a bicontinuous structure; (**c**) XRD profiles before and after swelling showing changes in crystalline/ordered structures. Adapted with permission from Ref. [18], © 2024 Wiley-VCH GmbH.

More recent non-isocyanate polyurethane (NIPU)/POSS hydrogels further show that nominal component hydrophobicity alone does not predict water uptake, because homogeneous POSS dispersion can preserve the accessibility of polymer hydration sites [41]. Water-vapor sorption and DSC measurements further reveal composition-dependent uptake and hydration-dependent thermal behavior of the absorbed water (Figure 3A) [41]. Total water content should therefore be considered together with method-specific information on water mobility and binding. Hydration state directly affects polymer dynamics. Water associated with polar segments generally increases local mobility and lowers the glass-transition temperature. In segmented PU, increasing hydration has been associated with a lower *T*_g_, altered dielectric relaxation, enhanced segmental mobility, changes in hard-domain stability, and accelerated stress relaxation [25,40,42].

### 3.2. Polymer–Water Interactions and Competitive Hydrogen Bonding

The hydrogen-bond network of segmented PU is established before hydration. Urethane and urea groups provide N–H donors and carbonyl acceptors, while polyether and polyester soft segments introduce additional acceptor sites. Competition between hard segment–hard segment and hard segment–soft segment hydrogen bonding contributes to the balance between hard-domain aggregation and phase mixing [12,14,17]. Hydration introduces an additional competing interaction: polymer–water hydrogen bonding.

Water can act as both a hydrogen-bond donor and acceptor and can interact with urethane/urea carbonyls, N–H groups, and polar soft-segment sites. In polytetramethylene glycol (PTMG)-based segmented PU, two-dimensional correlation Fourier-transform infrared (FTIR) spectroscopy showed that water initially interacted with free carbonyl groups and subsequently affected hydrogen-bonded carbonyls as hydration progressed [25]. This observation indicates that hydration does not disrupt the entire hydrogen-bond network uniformly but progressively modifies increasingly constrained molecular environments. Hydration-induced changes in carbonyl-, ether-, and urethane-related vibrational bands reported for polyether PU further support reorganization of local polymer–water and polymer–polymer interactions [43]. Related evidence is provided by NIPU/POSS hydrogels, in which hydration-induced shifts in the carbonyl and N–H bands indicate redistribution of hydrogen bonding toward polymer–water interactions (Figure 3B(a)) [41]. This redistribution is accompanied by pronounced changes in segmental dynamics: the glass-transition temperature decreases rapidly with initial water uptake, corresponding to a plasticization-dominated regime, whereas at higher hydration the polymer dynamics enter a slaving regime in which segmental mobility becomes increasingly coupled to the dynamics of bulk-like hydration water (Figure 3B(b)) [41]. More generally, solvation of hydrophilic segments and competition with pre-existing polymer–polymer associations can increase soft-phase and interfacial mobility, lower *T*_g_, weaken some physical junctions, and accelerate stress relaxation [25]. Crosslinked polyether PU networks similarly show enhanced H_2_O–urethane interactions and increased cooperative segmental mobility during hydration [42]. These effects, however, should not be generalized as a universal sequence of hydrogen-bond disruption followed by softening. Selective solvation can also alter phase compatibility and permit new intermolecular associations after segmental migration. The structural outcome therefore depends on composition, hydration level, network constraints, and the initial microphase state.

### 3.3. Water-Induced Microphase Separation and Microphase Reconstruction

Three processes should be distinguished when discussing hydration-dependent PU morphology. Intrinsic microphase separation refers to the soft/hard-segment organization already present before hydration as a result of thermodynamic incompatibility and specific intermolecular interactions [12,13]. Hydration-induced microphase reconstruction describes changes in the size, composition, connectivity, or interfaces of pre-existing domains after water uptake. Water-induced microphase separation should be reserved for cases in which selective hydration drives initially more mixed components toward newly developed compositional segregation or phase connectivity. These distinctions are essential because uniform swelling of an existing morphology does not by itself constitute new microphase separation [13,18,24,25].

Hydration-induced reconstruction arises from the nonuniform distribution of water across the pre-existing microphase structure. Preferential solvation of soft-segment-rich and interfacial regions increases local mobility, whereas cohesive hard domains remain more resistant to penetration and rearrangement. In segmented PU elastomers, this contrast is reflected in increased soft- and interfacial-region mobility, partial disruption of relatively loose hard domains, and accelerated stress relaxation [25]. Small-angle X-ray scattering (SAXS) and relaxation measurements therefore demonstrate that molecular-scale solvation can propagate to mesoscale organization and macroscopic viscoelastic behavior. Nevertheless, these observations primarily support hydration-induced perturbation or reconstruction of an existing microphase structure rather than formation of a completely new phase-separated morphology. Related wet-state surface studies also demonstrate hydration-driven segmental redistribution. In PUU, in-liquid atomic force microscopy (AFM) revealed pronounced rearrangement of near-surface microphase morphology during hydration [36,44], while PEG-containing segmented PU showed preferential migration of hydrophilic segments toward the polymer/water interface [45]. These observations confirm that hydration can drive local compositional redistribution, but near-surface restructuring should not be assumed to represent synchronous bulk evolution. This distinction is examined further in Section 3.4.

Water-induced microphase separation represents a distinct case in which hydration itself drives the formation of new compositional segregation. When sufficient hydrophilic–hydrophobic contrast and segmental mobility are present, selective solvation can reorganize initially mixed components into compositionally distinct domains during water exposure [46]. A direct hydrogel example was reported for PEG-PU/PCL-PU blends containing hydrophilic PEG-PU and hydrophobic PCL-PU. After swelling, differences in water affinity drove in situ phase separation, and an appropriate composition produced an interconnected bicontinuous morphology stabilized by multiple hydrogen bonds (Figure 3C) [18]. Three-dimensional structural reconstruction, XRD, SAXS, and related analyses supported the formation of new mesoscale organization rather than simple geometric expansion of the dry morphology [18].

The significance of the interconnected bicontinuous PEG-PU/PCL-PU morphology is consistent with the broader phase-separated-hydrogel framework, in which mesoscale connectivity between polymer-rich and solvent-rich regions can regulate mechanical and dynamic behavior [24]. For PU hydrogels, PU-specific evidence should establish a causal sequence in which selective hydration redistributes intermolecular interactions, promotes segmental migration, and ultimately alters microphase organization. Depending on composition and network constraints, this sequence may lead to increased phase mixing, disruption of pre-existing domains, reconstruction of existing interfaces, or formation of new phase-separated structures. Microphase evolution should therefore be treated as a system-dependent response to hydration rather than as an inevitable consequence of water uptake. Figure 3 summarizes representative multiscale evidence linking water uptake and water-state behavior to hydrogen-bond redistribution, segmental mobility, and ultimately hydration-driven microphase organization in polyurethane-based systems.

### 3.4. Surface–Bulk Structural Differences Under Hydrated Conditions

Hydration-induced reconstruction can differ substantially between the surface and bulk because the polymer/water interface is directly governed by interfacial free-energy minimization and preferential segmental exposure. Hydration-induced surface reconstruction has been examined in segmented PUU biomaterials intended for biological or blood-contacting interfaces using in-liquid AFM, AFM-based nanomechanical measurements, and protein/platelet interaction assays [36,47,48]. These measurements revealed time-dependent rearrangement of near-surface microdomains during hydration, accompanied by changes in local nanomechanical response and protein or platelet interactions. Related measurements indicated that some reconstructed surface structures did not immediately recover upon dehydration [36,48]. This behavior is not universal: in segmented PU containing strongly hydrophilic PEG segments, hydration can instead promote migration of hydrophilic components toward the water interface [45]. The direction of surface reconstruction therefore depends on composition, initial morphology, and interfacial energetics. Surface reconstruction should not be directly extrapolated to the bulk. AFM primarily probes near-surface morphology and local mechanics, whereas bulk evolution is additionally constrained by water diffusion, domain connectivity, and network topology. Bulk-sensitive measurements show that absorbed water can alter segmental dynamics, perturb relatively loose hard domains, and accelerate stress relaxation within segmented PU [25]. Reliable interpretation therefore requires a clear distinction between interface-driven surface restructuring from diffusion- and network-controlled bulk restructuring.

### 3.5. Wet-State and In Situ Multiscale Characterization

No single characterization method can establish the complete hydration–structure pathway in PU hydrogels. Water-state measurements, spectroscopy, microscopy, scattering, and mechanical analysis probe different length and timescales and should therefore be interpreted as complementary evidence. DSC and dielectric spectroscopy characterize thermodynamic water populations and molecular mobility, while changes in *T*_g_ and relaxation behavior provide information on hydration-induced plasticization [40]. FTIR/Raman spectroscopy can identify changes in local hydrogen-bonding environments, whereas solid-state NMR provides complementary information on chemical environments and segmental mobility; hydration-enhanced mobility in amorphous PU regions has been observed by ^13^C solid-state NMR [49]. Structural evolution requires spatially resolved evidence. Wet-state AFM can directly monitor near-surface morphology and local mechanical changes [36,47], whereas SAXS/WAXS and XRD are better suited to probing changes in bulk domain dimensions, periodicity, and ordering [13,25]. Ex situ SEM or TEM should be interpreted cautiously because dehydration, freezing, staining, or drying can alter water-rich morphologies and interdomain dimensions [50]. Consequently, neither spectroscopic shifts nor morphological changes obtained after drying should alone be treated as proof of hydration-induced bulk microphase reconstruction. Table 1 summarizes the corresponding evidence boundaries. Establishing how water penetration leads to hydrogen-bond redistribution, changes in segmental dynamics, subsequent microphase evolution, and finally network response requires mutually constraining measurements acquired, ideally, along the same hydration timeline. In particular, the formation of new water-induced phase separation requires evidence for newly developed compositional segregation or connectivity and cannot be inferred solely from increased domain spacing, swelling, or stress relaxation.

### 3.6. Mechanistic Synthesis: Hydration as a Structural Regulator

Taken together, hydration-mediated structural evolution in PU is governed by coupling among selective water uptake, intermolecular interactions, segmental mobility, and microphase constraints. In some systems, water acts predominantly as a plasticizer and hydrogen-bond competitor, increasing molecular mobility, perturbing relatively weak hard domains, and accelerating stress relaxation [25]. In others, appropriately designed hydrophilic–hydrophobic heterogeneity and network constraints allow selective hydration to promote phase redistribution or generate new bicontinuous organization [18]. Similar water contents can therefore give rise to markedly different structural responses.

Hydration should consequently not be described through a universal pathway of either “water-induced softening” or “water-induced strengthening.” Its effect depends on molecular composition, the initial microphase state, network topology, hydration level, and observation timescale. When increased mobility occurs without loss of the principal load-bearing structures, hydration can facilitate reversible reorganization; when physical junctions are excessively disrupted, softening and accelerated relaxation dominate; and when selective solvation drives favorable mesoscale reconstruction, mechanical reinforcement may emerge [18,25,46]. This competition among structure-dependent responses provides the basis for the dynamic-network behavior discussed in Section 4.

## 4. Dynamic Interactions and Network Reconstruction

Hydration-mediated microphase changes influence macroscopic behavior through the kinetics of reversible network rearrangement. In PU hydrogels, the relevant parameters are not only bond strength, but also junction lifetime, spatial distribution, and the segmental mobility required for exchange or reassociation. Because hydration and soft/hard-segment heterogeneity regulate local mobility and interaction accessibility, dynamic-network behavior is inherently coupled to both water state and microphase structure. Consistent with studies of dynamic covalent PU networks, self-healing and structural recovery therefore depend on matching bond-exchange kinetics with polymer-chain mobility rather, than on the presence of reversible bonds alone [19].

### 4.1. Hydrogen Bonding and Hydrophobic Association

Hydrogen bonding provides an intrinsic dynamic component of segmented PU because hard-segment-associated interactions undergo continuous association and dissociation [17]. In hydrated networks, their mechanical contribution therefore depends on whether dissociation during deformation can be followed by sufficiently rapid reassociation during recovery. More broadly, supramolecular hydrogels demonstrate that directional noncovalent interactions can support reconstructable networks [56], while cooperative hydrogen bonding can increase the effective lifetime and mechanical contribution of individually weak interactions [9,57]. In PU-based systems, rapid recovery has been demonstrated in double-network hydrogels composed of a PU network and a dipole–dipole and hydrogen-bonding interaction-reinforced (DHIR) network [54], whereas water-induced bicontinuous PU hydrogels illustrate how multiple hydrogen bonds can couple local deformation-induced mechanical energy dissipation with mesoscale phase connectivity [18]. Hydrophobic association provides an additional hydration-sensitive physical junction because exposure of hydrophobic segments to water is energetically unfavorable, promoting their aggregation into transient domains that can act as physical crosslinks. In amphiphilic PEG/PCL-containing PU hydrogels, the balance between hydrated PEG-rich segments and hydrophobic PCL-rich associations therefore varies with composition and influences network organization and swelling [58]. Amphiphilic PEG/PCL-containing PU hydrogels exhibit composition-dependent hydration and association behavior [58], while oligo(ethylene glycol) (OEG)-containing PU undergoes reversible hydrophilic–hydrophobic switching accompanied by changes in aqueous swelling [59]. Hydrogen bonding and hydrophobic association are therefore most appropriately considered as coupled, water-dependent contributors to effective crosslinking and microphase organization.

### 4.2. Metal–Ligand, Ionic, and Polyphenol-Mediated Noncovalent Interactions

Metal–ligand and ionic interactions provide reversible junctions whose strength and exchange kinetics can complement hydrogen bonding in PU hydrogels. For example, in chemically crosslinked PU/tannic acid (TA) hydrogels, tannic acid forms multiple reversible hydrogen bonds with PU chains [55]. In Fe^3+^-containing phenol–carbamate PU hydrogels, Fe^3+^ coordinates with polyphenol groups to form a secondary reversible network that couples dynamic crosslinking with photothermal deformation [35]. In waterborne-polyurethane-reinforced zwitterionic conductive hydrogels, ionic components additionally modify charge screening, osmotic pressure, and hydration [60]. The contribution of these noncovalent interactions to macroscopic behavior therefore depends on their exchange kinetics together with local hydration and polymer mobility, rather than on interaction strength alone.

### 4.3. Dynamic Covalent Bonds and Exchange Timescales

Dynamic covalent bonds combine persistent network connectivity with time-dependent topological rearrangement and therefore occupy an intermediate kinetic regime between permanent crosslinks and rapidly exchanging supramolecular junctions [19]. Disulfide chemistry is particularly common in dynamic PU networks, but its exchange kinetics are strongly dependent on molecular structure and environmental conditions. Aromatic disulfide metathesis can proceed at room temperature in appropriately designed poly(urea–urethane) networks [61], whereas other PU systems exhibit thermally or otherwise activated healing behavior [62]. Conversely, suitably designed waterborne PU networks containing exchangeable disulfides can also undergo room-temperature self-healing [63]. In PU hydrogels, disulfide-containing tetra-PEG networks combine covalent connectivity with redox-responsive degradation [33], while dual-dynamic networks couple disulfide exchange with faster hydrogen-bond reorganization [53]. PEG-based PU hydrogels containing tertiary amines similarly combine dynamic disulfides with room-temperature healing and pH-dependent swelling [32]. In acylhydrazone-containing PU hydrogels, reversible acylhydrazone exchange enables covalent-network rearrangement and contributes to self-healing [31], whereas imine-based hydrogels provide broader mechanistic evidence for exchange-assisted interfacial reconnection and injectability [64].

Dynamic exchange must also be distinguished from irreversible or stimulus-induced bond cleavage. Disulfide exchange reorganizes network topology without necessarily reducing connectivity, whereas reductive cleavage decreases effective crosslink density and can promote swelling or degradation. Combining relatively rapid supramolecular interactions with slower covalent exchange can therefore separate the timescales of energy dissipation, interfacial closure, and longer-term structural recovery [31,53,65].

### 4.4. Spatial Coupling Between Microphase Structure and Dynamic Bonds

Dynamic bonds act within spatially heterogeneous PU environments rather than in a uniform matrix. Their local concentration, mobility, and exchange kinetics therefore depend on whether they are located in soft segments, hard domains, or interphase regions. Microdomain-confined disulfides provide a representative example: confinement within hard-segment-rich regions can preserve load-bearing cohesion while allowing local reorganization [66]. Changing disulfide content can also modify phase morphology and molecular mobility [67], while aromatic disulfides can alter hard-domain packing and hierarchical hydrogen bonding during deformation [68]. In hydrated PU, this coupling becomes bidirectional. Water can increase mobility in soft and interfacial regions while weakening some physical junctions, whereas selective hydration can also promote microdomain rearrangement [18,25]. Consequently, the effect of a dynamic bond cannot be inferred from its concentration alone; its spatial location within the microphase structure and the local hydration state are equally critical determinants of its behavior.

### 4.5. Molecular Mobility, Stress Relaxation, and Interfacial Self-Healing

For PU hydrogels, self-healing requires more than re-formation of reversible bonds. After damage, the opposing interfaces must first re-establish contact, followed by segmental migration or interdiffusion and re-formation of load-bearing interactions. Broader supramolecular and dynamic-imine hydrogel studies support the same mechanistic requirement: microscopic rebonding produces macroscopic recovery only when bond dynamics are compatible with polymer mobility [9,57,64,69].

In PU, these processes are strongly affected by hydration and hard-domain organization. Water absorption can increase soft-phase and interfacial mobility and accelerate stress relaxation [25]. Moisture-exposed PUU provides PU-specific mechanistic evidence that plasticization and hydrogen-bond redistribution facilitate molecular mobility during healing [51]. Because this study concerns moisture-conditioned PUU rather than an equilibrium PU hydrogel, its results are used here to support the qualitative mechanism and are not treated as directly transferable quantitative hydrogel data. Multiple-timescale designs can partially decouple interfacial closure from structural recovery: hydrogen bonds may reorganize faster than disulfide exchange [65], reversible physical interactions can accelerate recovery in PU double networks [54], and microdomain confinement can retain load-bearing structures during local rearrangement [66]. PU hydrogels containing acylhydrazone/hydrogen-bond or disulfide/hydrogen-bond combinations provide corresponding hydrogel examples [31,32,53].

Network recovery can thus be viewed as a sequential process involving reversible-bond reorganization, segmental migration, microdomain adjustment, and restoration of network continuity.

## 5. Microphase- and Dynamic-Network-Mediated Strengthening, Toughening, and Fatigue Resistance

Mechanical performance in PU hydrogels arises from the coupling of soft-phase extensibility, hard-domain load transfer, energy dissipation through reversible interactions, and hydration-dependent network dynamics. Classical double-network, ionic–covalent, and polyampholyte hydrogels established general strategies for combining stable load-bearing structures with dissipative components [6,7,8]. In segmented PU hydrogels, these mechanisms are further regulated by microphase hierarchy and dynamic junctions, making strength, toughness, recovery, and cyclic performance dependent on both structural organization and hydration state.

### 5.1. Mechanical-Property Evaluation Framework

Mechanical metrics should be separated according to the failure process they describe. Modulus characterizes small-strain stiffness; tensile or compressive strength and elongation at break describe maximum load and deformability; toughness is the area under an unnotched stress–strain curve; and fracture energy quantifies crack propagation under a specified fracture geometry. Reversible interactions can enlarge hysteresis and apparent toughness but may also generate residual strain when recovery is incomplete [8,10]. Fatigue is distinct from both single-cycle toughness and unnotched cyclic stability. When fatigue thresholds or crack-growth rates are discussed, precracked specimens and energy-release-rate-based protocols are required [11]. Cross-study comparisons should therefore specify hydration state, equilibration time, specimen geometry, temperature, loading rate, and intercycle interval because these variables directly affect relaxation and structural recovery in dynamic PU hydrogels.

To provide a more quantitative comparison among representative PU hydrogels, Table 2 summarizes selected physical and mechanical properties reported under hydrated or equilibrium-swollen conditions, including hydration or swelling metrics, modulus, strength, elongation at break, toughness or fracture resistance, and cyclic-performance indicators where available. The available data demonstrate a broad property space, ranging from highly swollen, relatively compliant PU–PEG networks to mechanically robust phase-separated and dual-crosslinked PU hydrogels. Because the reported values depend strongly on water content, swelling state, network composition, and testing mode, hydration information is included whenever available. The tabulated data should therefore be interpreted as representative system-specific benchmarks rather than as an absolute ranking of material performance.

The comparison highlights that high hydration does not correspond to a unique mechanical regime in PU hydrogels. Highly swollen PU–PEG or PEG–POSS networks can span kPa-to-MPa stiffness ranges, whereas dual-crosslinked or microphase-engineered systems can retain MPa-level strength and substantial fracture resistance under hydrated conditions. These differences reflect not only chemical composition but also crosslinking architecture, microphase organization, and hydration state, reinforcing the need to interpret numerical performance together with the corresponding network structure and testing conditions. Selected dynamic or dual-crosslinked PU hydrogels also exhibit substantial cyclic recovery; however, quantitative crack-growth-based fatigue data remain limited, and cyclic loading–unloading stability should not be interpreted as equivalent to a true fatigue threshold.

### 5.2. Sacrificial Interactions and Energy Dissipation Through Reversible Junctions

A common strategy for toughening hydrogels is to introduce structures that dissipate energy before failure of the primary load-bearing network. In conventional double-network hydrogels, dissipation originates largely from irreversible damage to the first network [6], whereas ionic–covalent and some reversible networks demonstrate that sacrificial interactions can dissociate during loading and subsequently reform [7,8,10].

**Figure 4:**
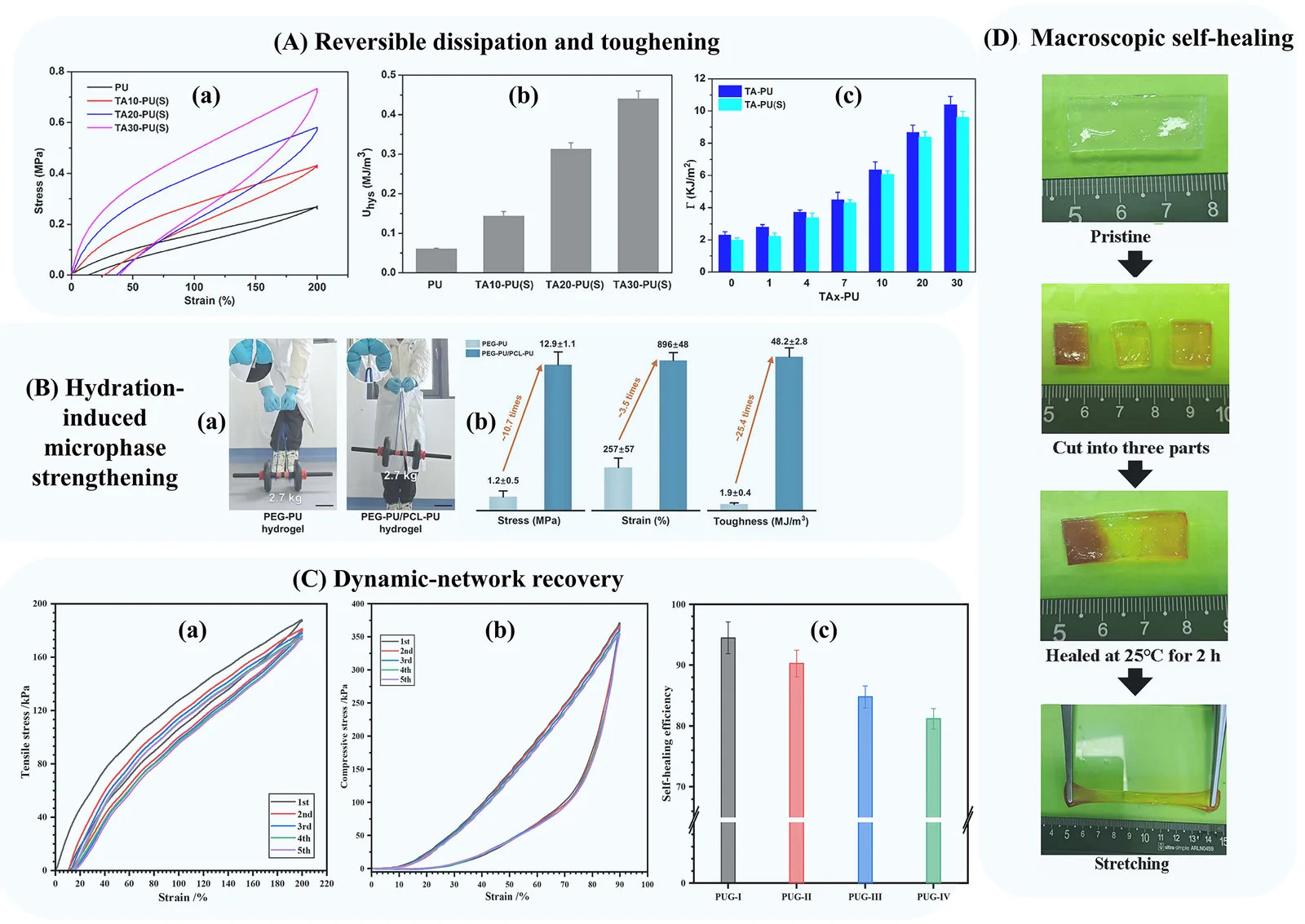
Representative experimental evidence for microphase- and dynamic-network-mediated mechanical regulation in polyurethane hydrogels. (**A**) Energy dissipation and toughening enabled by reversible PU–TA interactions, including loading–unloading behavior, hysteretic energy density, and fracture energy. Reproduced from Ref. [55]. (**B**) Hydration-induced microphase strengthening of PEG-PU/PCL-PU hydrogels through in situ water-induced microphase separation, including macroscopic load-bearing and mechanical-property comparisons. Reproduced with permission from Ref. [18]. Copyright 2024, Wiley-VCH GmbH. (**C**) Cyclic tensile/compressive recovery and self-healing efficiency of disulfide-containing PEG-based PU hydrogels. (**D**) Macroscopic cut–heal–stretch demonstration of network reconstruction. (**C**,**D**) Reproduced from Ref. [32].

PU hydrogels provide several structural levels for reversible dissipation, including urethane/urea hydrogen bonds, hydrophobic associations, interfacial interactions, and additional polyphenol, ionic, or coordination junctions. Hard-segment-rich domains themselves, however, should not be generically regarded as sacrificial bonds; they contribute to dissipation only when their internal or interfacial structures undergo load-induced reversible rearrangement. In dual-crosslinked PU/TA hydrogels, multiple PU–TA hydrogen bonds substantially increased hysteretic dissipation and fracture resistance under swollen conditions (Figure 4A) [55]. At 200% strain, the dissipated energy of TA30–PU approached 440 kJ·m^−3^, approximately sevenfold that of the neat PU hydrogel, while its equilibrium-swollen fracture energy remained approximately 9.6 kJ·m^−2^ [55]. These results demonstrate that reversible secondary crosslinks can increase energy dissipation while maintaining substantial wet-state resistance to crack propagation.

The mechanical benefit of dissipation nevertheless depends on recovery kinetics. Large hysteresis contributes to repeated-load performance only when the relevant junctions and local structures recover on a timescale comparable to the loading interval. Hydration therefore couples energy dissipation and recovery by simultaneously regulating interaction stability and segmental mobility.

### 5.3. Microphase-Structure-Mediated Strengthening and Toughening

Segmented PU provides an intrinsic mechanical hierarchy in which soft phases accommodate large deformation, whereas hard-segment-rich domains provide physical crosslinking and load transfer. Mechanical behavior consequently depends on domain size, connectivity, interfacial cohesion, and phase morphology rather than on hard-segment fraction alone [70].

Water-induced bicontinuous PU hydrogels provide a direct example of microphase-mediated mechanical reinforcement (Figure 4B). In PEG-PU/PCL-PU systems, selective hydration generates an interconnected bicontinuous morphology stabilized by multiple hydrogen bonds [18]. The optimized hydrogel exhibited a fracture strength of 12.9 MPa, toughness of 48.2 MJ·m^−3^, and fracture energy of 2435 J·m^−2^ [18]. Combined with three-dimensional structural reconstruction and scattering evidence, these results support a mechanism in which a continuous polymer-rich phase maintains load transfer, while hydrated regions, interfaces, and reversible hydrogen bonds accommodate deformation and energy dissipation.

Hydration-induced reinforcement is nevertheless system-dependent. Swelling-induced hardening has also been reported in some PU hydrogel architectures [71], whereas strain-induced microphase separation in non-PU supramolecular hydrogels provides a broader mechanistic analogue [72]. Together, these observations indicate that phase reorganization can modify load transfer and dissipation, although the direction and magnitude of the resulting mechanical response depend on composition, hydration state, and network constraints.

### 5.4. Double, Multiple, and Heterogeneous Networks

Network topology provides a second level of mechanical regulation beyond microphase structure. Classical double networks combine a dissipative network with a more extensible load-bearing network [6], while ionic–covalent systems introduce reversible junctions that improve recoverability [7]. PU hydrogels can implement similar functional partitioning through multiple architectures, including PU–gelatin double networks [73], chemically/physically dual-crosslinked PU/TA hydrogels [55], heterogeneous single-network PU hydrogels [34], and covalently crosslinked degradable PU hydrogels [29]. These systems are more usefully compared by mechanical function than by the nominal number of networks. Stable connections maintain shape and transfer load, whereas reversible or locally damageable structures broaden the dissipation zone and permit recovery. Because hard domains already constitute a physical network in segmented PU, additional covalent and dynamic junctions should be designed to complement this intrinsic hierarchy rather than merely increase network complexity.

### 5.5. Cyclic Recovery Versus Fatigue Resistance

Long-term cyclic performance depends on the competition between deformation-induced damage and structural recovery. Reversible junctions can retain their dissipative function when recovery occurs faster than the interval between loading events; otherwise, residual strain and local structural damage can progressively accumulate. High monotonic toughness therefore does not establish resistance to cyclic crack growth, which requires explicit fatigue-fracture measurements [11]. Hierarchical reinforcing structures provide a general strategy for combining dissipation with recovery [74], while PU-specific approaches include dual-dynamic networks and microdomain-confined reversible interactions [31,66].

Disulfide-containing PEG-based PU hydrogels provide a representative example of cyclic recovery and post-damage reconstruction (Figure 4C,D) [32]. PUG-II retained 92.7% of its initial tensile stress after five consecutive loading–unloading cycles at 200% strain and exhibited a residual strain of 15.9%, while five successive compression cycles to 90% strain produced nearly overlapping stress–strain curves (Figure 4C) [32]. The same study demonstrated efficient room-temperature self-healing across the PUG series, with the highest healing efficiency reaching 94.5% after 6 h, and macroscopic cut–heal–stretch behavior further demonstrated the restoration of interfacial continuity (Figure 4D) [32]. These measurements demonstrate cyclic stability, self-recovery, and post-damage network reconstruction, but they do not directly determine a fatigue threshold or crack-growth rate.

Fatigue-resistant design therefore requires explicit consideration of loading and recovery timescales. Slowly recovering junctions can behave as effectively irreversible damage under rapid cycling, whereas excessively fast exchange may increase stress relaxation and reduce persistent load-bearing capacity. Cyclic hysteresis, interval-dependent recovery, and precracked fatigue tests should consequently be regarded as complementary measures of distinct aspects of mechanical durability.

### 5.6. Trade-Offs Among Hydration, Strength, Toughness, and Recovery

Hydration introduces a kinetic–structural trade-off because it simultaneously regulates segmental mobility and the integrity of physical load-bearing structures. At relatively low hydration, hard-domain cohesion and polymer–polymer associations can remain well preserved, but limited molecular mobility may restrict reversible rearrangement. Increasing hydration can promote stress relaxation, segmental migration, and network reorganization, whereas excessive hydration may weaken physical junctions and increase creep, consistent with hydration-dependent changes in molecular dynamics and relaxation observed in segmented PU [25]. PU hydrogels can nevertheless exhibit design-dependent mechanical responses rather than simple monotonic water-induced softening, as illustrated by thermoresponsive tough PU hydrogels [75], crosslink-dependent mechanical regulation [29,76], water-induced bicontinuous reinforcement [18], and swelling-induced hardening [71].

**Figure 5:**
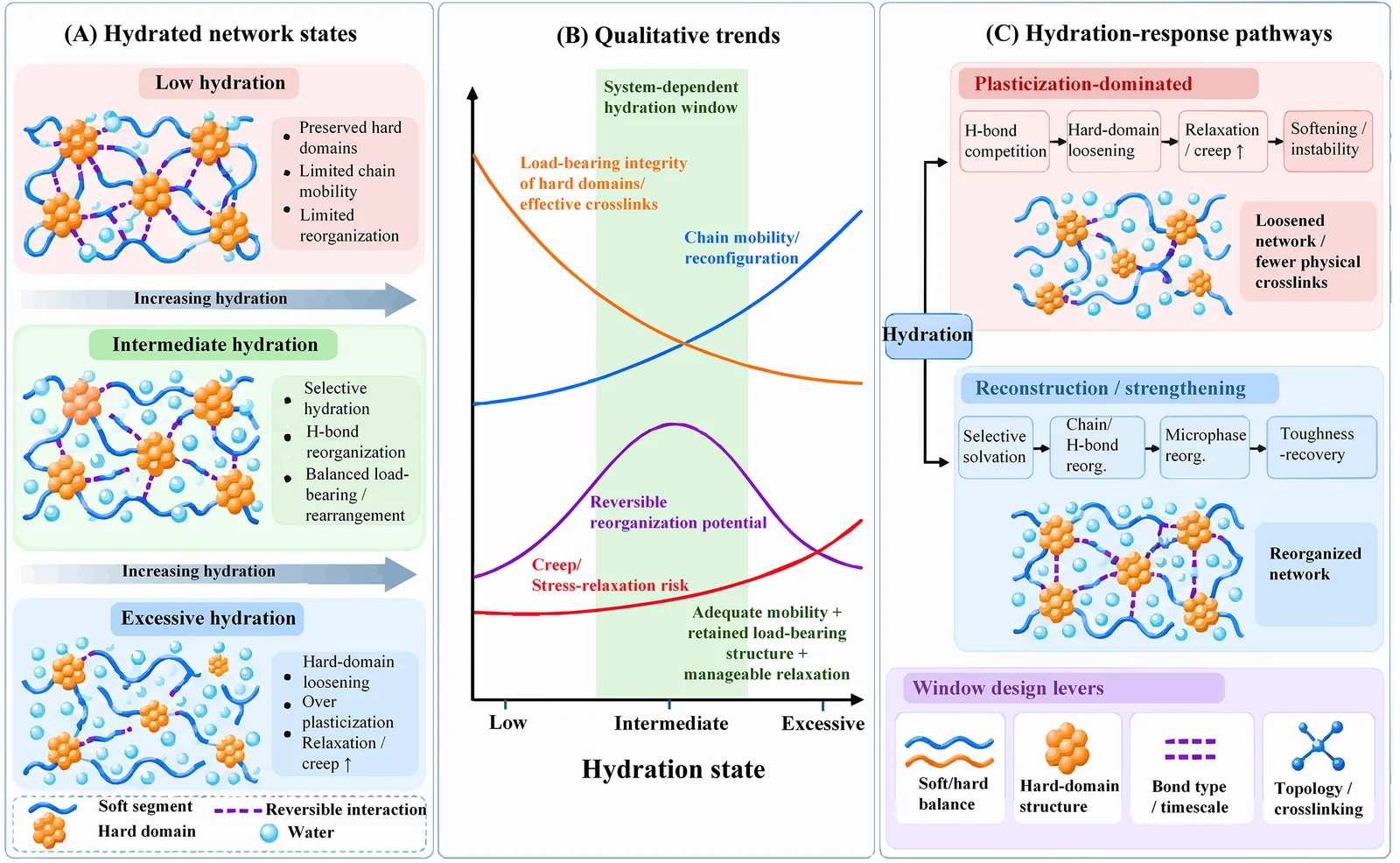
Conceptual framework of the system-dependent hydration window in polyurethane hydrogels. (**A**) Representative network states across increasing hydration, illustrating concurrent changes in segmental mobility and load-bearing domain integrity. (**B**) Qualitative competition among load-bearing integrity, segmental mobility, reversible reorganization, and creep/stress-relaxation risk. The intermediate region represents a system-specific hydration–network operating window rather than a fixed range of total water content. (**C**) Alternative hydration-response pathways dominated by plasticization/relaxation or reconstruction-mediated reinforcement, together with the principal molecular and network variables regulating the hydration window. All trends are schematic and system-dependent rather than universal quantitative relationships.

On this basis, we propose a system-dependent hydration window as a testable framework rather than a universal optimum (Figure 5). Importantly, this window represents a coupled hydration–network state rather than a fixed range of total water content. It denotes conditions under which sufficient water-mediated segmental mobility for reversible reorganization coexists with persistent load-bearing domains and acceptable creep or stress relaxation. Its position and width depend on soft/hard-segment chemistry, water state, microphase organization, network topology, dynamic-interaction kinetics, and the relevant loading timescale. As illustrated in Figure 5, hydration-dependent behavior may follow a plasticization/relaxation-dominated pathway or, in appropriately designed systems, a reconstruction-mediated reinforcement pathway. Direct PU-hydrogel evidence supports water-induced microphase organization and swelling-induced hardening [18,71], whereas related aqueous PU systems provide additional mechanistic evidence for hydration-induced molecular rearrangement and phase-dependent mechanical changes [46]. These pathways should therefore be regarded as system-dependent qualitative relationships rather than universal quantitative functions.

Experimental validation of the proposed hydration window requires systematic control of hydration together with measurements of water state, microphase structure, stress relaxation, structural recovery, and fatigue behavior. Mechanical design of PU hydrogels can consequently be reframed from optimizing an isolated strength or toughness metric to coordinating hydration state, load-bearing integrity, microphase organization, reversible-network dynamics, and loading timescale. The same framework also provides a mechanistic connection to stimuli responsiveness because temperature, pH, ionic conditions, and redox environments can alter polymer–water interactions and shift the network between different structural states.

## 6. Hydration-Coupled Stimuli Responsiveness and Injectability

External stimuli can regulate PU hydrogels by shifting polymer–water affinity, charge state, hydrophobic association, or dynamic-bond stability. These molecular changes alter hydration, effective crosslink density, segmental mobility, and phase organization, producing macroscopic changes in swelling, rheology, transport, or network state. The following sections therefore focus on how different stimuli converge on this shared hydration–network coupling rather than treating each response as an independent function.

### 6.1. Thermoresponsiveness and Sol–Gel Transitions

Thermoresponsive PU hydrogels are governed by temperature-dependent competition between polymer hydration and intermolecular association. PEO–poly(propylene oxide) (PPO)–PEO-based PU systems exhibit reverse thermoresponsive behavior associated with changes in segmental hydration and hydrophobic association [77], while OEG-containing PU undergoes reversible hydrophilic–hydrophobic switching [59]. In PEG/PCL-containing poly(ester-ether urethane) hydrogels, temperature-dependent swelling can likewise be regulated by the relative PEG/PCL content and the degree of covalent crosslinking [78]. Polyether-based PU can also exhibit composition-tunable lower critical solution temperature (LCST) behavior [79]. At sufficiently high polymer concentrations, thermally promoted intermolecular association can produce a macroscopic sol–gel transition, as demonstrated for poloxamer 407 (P407)-derived poly(ether urethane) systems [80]. More recent studies further show that PEG molecular weight and hydrophobic structure can systematically regulate the transition temperature and gel formation [81]. Related biodegradable PU systems have been used for in situ gelation, cell encapsulation, and drug delivery [80,82,83]. Thermoresponsive design therefore requires coordinated control of transition temperature, gelation kinetics, hydration, and post-gelation stability.

### 6.2. pH and Ionic Responsiveness

pH responsiveness arises primarily from changes in ionization that modify electrostatic interactions, osmotic pressure, and polymer hydration. In PEG-based PU hydrogels containing tertiary amines, reversible protonation produces pronounced pH-dependent swelling while retaining disulfide-mediated dynamic-network behavior [32]. Carboxyl-containing thermoresponsive PU hydrogels similarly couple pH-dependent hydration with network changes and drug release [84]. 2,2-bis(hydroxymethyl)propionic acid (DMPA)-containing supramolecular PU hydrogels have been used for pH-triggered nanocarrier release [85], whereas anthocyanin-containing PU hydrogels convert pH changes into a visible response [86]. Ionic conditions can additionally modify charge screening, hydration, or coordination junctions, as illustrated by Fe^3+^–polyphenol PU networks [35]. pH-responsive behavior should therefore be interpreted under defined ionic conditions because protonation, ionic strength, and coordination can alter the hydrated network simultaneously.

### 6.3. Redox, Photo-, and Multistimuli Responsiveness

Redox and photoresponses do not necessarily originate from hydration, but their effects can propagate through changes in network connectivity and water distribution. In disulfide-containing tetra-PEG PU hydrogels, reductive S–S cleavage decreases effective crosslink density and promotes network degradation [33]; this process should be distinguished from dynamic disulfide exchange, which can reorganize topology without necessarily reducing connectivity. Light-responsive PU hydrogels can operate through distinct molecular mechanisms. In azobenzene/cyclodextrin-containing PU hydrogels, light-induced azobenzene photoisomerization modulates host–guest interactions, while disulfide bonds provide an independent redox-responsive motif [87]. In Fe^3+^–polyphenol PU networks, near-infrared irradiation is converted into heat, and the resulting temperature increase drives macroscopic deformation [35]. Related non-PU injectable photothermal hydrogels provide broader mechanistic support for this photothermal pathway but are cited here only as mechanistic analogues [88]. Multistimuli PU systems further demonstrate that temperature, pH, redox conditions, and light can converge on polymer–water affinity, effective crosslink density, and network dynamics [33,84,87,89]. Their design is therefore governed more by response sequence and kinetic compatibility than by the number of incorporated responsive motifs.

### 6.4. Injectability: Thermally Induced Gelation and Shear-Induced Network Reconstruction

Injectability in PU hydrogels is achieved mainly through two network transitions. In thermally induced gelation, a flowable precursor forms a physical network after administration, as demonstrated by P407-derived poly(ether urethane) systems [80], biodegradable thermoresponsive PU used for cell encapsulation and bioprinting [82], and PU/mesoporous-glass formulations for localized delivery [83]. Alternatively, shear can temporarily disrupt reversible junctions and reduce flow resistance, followed by rapid network recovery once the shear is removed; poly(ether urethane)/α-cyclodextrin hydrogels provide a representative supramolecular example [90]. Injectability should therefore be evaluated as a kinetic transition between a flowable state and a stable post-injection network rather than by needle passage alone. Thermogelling systems require appropriate gelation temperature, gelation time, and post-gel modulus, whereas shear-thinning systems require characterization of viscosity under shear and modulus recovery after shear cessation. Systems combining both mechanisms additionally require verification that injection does not induce irreversible phase separation or network damage.

### 6.5. Hydration–Network Coupling Framework for Stimuli Responsiveness

Although temperature, pH, ionic conditions, redox reactions, and light initiate responses through different molecular events, their effects converge on a limited set of network variables. Changes in molecular interactions or connectivity alter polymer–water affinity, effective crosslink density, and segmental mobility, which subsequently regulate micellization, microphase organization, rheology, mass transport, degradation, or actuation. Hydration is therefore best regarded as a coupling variable rather than the universal origin of stimuli responsiveness. This distinction is particularly important for redox cleavage and photothermal conversion, in which the initiating event is chemical or energetic, but the macroscopic response can be further amplified by subsequent redistribution of water and changes in network state.

## 7. Interfacial Reconstruction, Adhesion, and Anti-Adhesion in Aqueous Environments

Interfacial behavior represents the boundary-level manifestation of hydration–microphase–network coupling. In aqueous environments, competitive solvation and hydration-induced segmental redistribution can alter surface chemistry, local mechanics, and molecular contact. Interfacial water plays contrasting roles in wet adhesion and anti-adhesion. Wet adhesion generally requires partial displacement or reorganization of interfacial water so that adhesive groups can establish molecular contact with the substrate, whereas anti-adhesion relies on retention of a stable hydration layer that suppresses direct molecular contact. Both responses are therefore governed by the hydrated surface state rather than by dry-state surface chemistry alone.

### 7.1. Hydration-Induced Surface Reconstruction and Interfacial Consequences

Hydration can drive near-surface segmental redistribution and microdomain rearrangement in segmented PU, with the direction and extent of restructuring depending on soft/hard-segment chemistry and the initial microphase state [36,45,47]. Surface reconstruction has been investigated in hydrated segmented PUU biomaterials using in-liquid AFM and AFM-based nanomechanical measurements, together with assays of protein and platelet interactions [36,47,48]. These measurements showed time-dependent rearrangement of near-surface microdomains during hydration, accompanied by changes in local nanomechanical response and protein or platelet interactions. Surface-sensitive measurements should therefore be interpreted together with hydration history when relating PU microstructure to interfacial function.

### 7.2. Wet Tissue Adhesion

Robust wet adhesion requires three coupled processes: reorganization or displacement of interfacial water, formation of molecular connections across the interface, and sufficient bulk cohesion to resist crack propagation. In PU systems, catechol/polyphenol chemistry provides a representative route to this coupling. Lysine–dopamine-functionalized PU can undergo Fe^3+^- or oxidation-triggered gelation, allowing catechol-related interactions to contribute to both interfacial bonding and network cohesion [91]. Waterborne polyurethane (WPU)-crosslinked hydrogels provide another example in which chemical crosslinking and PU-rich microdomains support repeatable adhesion to diverse substrates, including a qualitative demonstration on animal muscle tissue [92]. WPU-reinforced zwitterionic conductive hydrogels further illustrate that adhesive behavior can coexist with mechanical and ionic functionality [60].

More generally, wet adhesion depends on the combined chemistry of interfacial bonds, topology of network–substrate connections, and mechanics of energy dissipation [93,94]. For PU hydrogels, increasing the density of adhesive groups is therefore not sufficient by itself: excessive crosslinking can limit conformal contact and segmental adaptation, whereas excessive hydration can weaken both molecular anchoring and bulk cohesion. Effective wet adhesion requires these interfacial and bulk processes to remain mechanically compatible.

### 7.3. Interfacial Toughness and Fatigue-Resistant Adhesion

Interfacial strength, interfacial toughness, and interfacial fatigue resistance quantify different aspects of adhesive failure and should be distinguished. A high maximum adhesive load does not necessarily imply strong resistance to crack propagation; single-cycle interfacial toughness depends on both interfacial bonding and bulk energy dissipation [93,94]. Stable network–substrate anchoring can increase the energy required for interfacial crack propagation [93]. Under repeated loading, however, the contribution from dissipative mechanisms may progressively decrease, so continued crack growth becomes increasingly governed by the intrinsic fatigue resistance of the bonded interface [95]. Accordingly, adhesion strength describes the maximum interfacial load, interfacial toughness quantifies the energy required for crack propagation during a single fracture event, and the interfacial fatigue threshold characterizes resistance to repeated crack advance. Test geometry is therefore critical: probe-pull or lap-shear tests primarily quantify maximum load, whereas peel or other fracture-based configurations are more appropriate for evaluating interfacial toughness [94]. Comparisons across studies should additionally account for substrate properties, specimen thickness, hydration state, loading rate, and failure mode.

For PU hydrogels, existing studies have more commonly demonstrated repeatable adhesion or unnotched cyclic mechanical stability than interfacial fatigue crack-growth resistance [92]. Direct determination of wet-state interfacial fatigue thresholds therefore remains limited. Long-term evaluation should combine cyclic interfacial loading with fracture-based measurements under sustained hydration to determine whether dynamic-bond recovery and hydration-induced structural reorganization can genuinely suppress interfacial crack propagation.

### 7.4. Antifouling, Anti-Tissue Adhesion, and Low Underwater Oil Adhesion

Antifouling, biological anti-adhesion, and low underwater oil adhesion share the use of interfacial hydration to suppress direct contact, but their performance criteria differ. PU–PEG hydrogels exploit strongly hydrated PEG-rich interfaces to reduce nonspecific protein and cellular interactions while retaining tunable mechanics [96], and degradable triol-crosslinked PU hydrogels similarly combine mechanical robustness with antifouling and anti-adhesive behavior [29]. Hydrophilicity alone, however, does not determine biological interfacial performance because microphase organization and hydration-induced surface reconstruction also influence local protein interactions [36,47]. The same interfacial-water principle extends to underwater oil repellency. Porous PU hydrogels can retain an aqueous interfacial layer that limits direct oil–polymer contact and thereby enables ultralow underwater oil adhesion for oil–water separation [97]. Thus, strong wet adhesion and anti-adhesion represent opposing outcomes of the same hydrated-interface problem: adhesion requires controlled removal or reorganization of interfacial water, whereas antifouling and underwater oil repellency require its stable retention.

## 8. From Structure–Property Relationships to Applications

The applications of PU hydrogels impose different combinations of hydration, mechanics, mass transport, degradation, interfacial behavior, and structural recovery. Rather than cataloging individual functions, this section considers representative applications as design problems in which the required service state defines the appropriate molecular architecture and network dynamics. The broad biomedical versatility of PU derives from its tunable chemistry, mechanics, degradation, and interfacial properties [20,21,22,98]. Within the framework developed above, application design can be approached by first defining the required property combination, then identifying the corresponding hydration and network state, and finally selecting the molecular and microphase architecture capable of generating that state.

### 8.1. Biodegradation and Biocompatibility

In biomedical PU hydrogels, degradation is coupled to hydration because water penetration governs the accessibility of hydrolytically, enzymatically, or stimulus-sensitive structures while network cleavage progressively alters mechanics and transport. Degradation behavior can be regulated through crosslink structure, microdomain organization, and cleavable chemistry, as illustrated by triol-crosslinked biodegradable PU hydrogels, microdomain-engineered dynamic PU networks, and light-degradable PUU hydrogel films [29,66,99]. The design objective is therefore not to maximize the degradation rate, but to achieve appropriate temporal matching among mass loss, mechanical retention, and the intended biological function. Biocompatibility further requires control of residual reactants, degradation products, and long-term interfacial responses [21,100]. Hydration, degradation, and mechanical stability should consequently be evaluated as coupled service-state variables rather than independent material properties.

### 8.2. Tissue Engineering and Regenerative Medicine

Tissue-engineering applications require simultaneous control of wet-state mechanics, mass transport, interfacial compatibility, and degradation [2,3]. The relative importance of these variables is tissue specific. Cartilage-related environments place particular demands on load bearing and long-term mechanical stability under high hydration [20], whereas spatially graded PU–alginate networks demonstrate how heterogeneous mechanics can be used to reproduce osteochondral transitions [101]. Neural interfaces, wound-healing systems, and cell-delivery platforms generally require relatively compliant hydrated environments together with efficient mass transport. Representative PU-based systems include PEG-containing PU coatings for neural interfaces [102], thermoresponsive PU hydrogels for cell encapsulation [82], and waterborne or composite PU hydrogels for wound-dressing applications [103,104,105,106]. A more useful design strategy is therefore to define the mechanical, hydration, transport, and degradation requirements of the target tissue first and subsequently select the corresponding soft/hard-segment composition and network architecture.

### 8.3. Drug Delivery and Injectable Therapies

Drug transport in PU hydrogels depends not only on total swelling but also on drug partitioning, polymer–drug interactions, molecular mobility, and degradation [1,58,107]. Consequently, similar equilibrium water contents can produce different release profiles when the local chemical and dynamic environments differ. Injectable PU systems add a second design requirement: the network must first establish rapid post-injection retention and subsequently permit controlled diffusion or degradation. Thermosensitive PU/mesoporous-glass formulations, hybrid responsive systems, and cyclodextrin-based supramolecular poly(ether urethane) (PEU) hydrogels illustrate complementary approaches to localized and sustained delivery [83,89,90,108]. Successful formulations must therefore match the injection/gelation timescale to the desired drug-release period rather than maximize loading or stimulus responsiveness independently [84,109].

### 8.4. 3D Bioprinting

3D bioprinting directly embodies the competing requirements of dynamic network reconstruction during processing and structural stability after deposition. During extrusion, PU hydrogels require appropriate shear-thinning behavior or reversible network disruption, whereas after deposition they must rapidly recover a continuous network and maintain long-term stability under hydrated conditions. Evaluation of PU bioinks should therefore extend beyond printing resolution to include post-shear network recovery, shape retention during long-term hydration, cytocompatibility, and the coordinated evolution of modulus and degradation during culture. From a structural-design perspective, the printing process and the subsequent service state impose two distinct requirements: processing requires rapid reversible network rearrangement to reduce flow resistance, whereas the post-printing culture state requires rapid network recovery followed by sufficient structural stability under sustained hydration. Representative systems include thermoresponsive degradable PU, PU/protein composite systems, and PU–gelatin double networks used for cell encapsulation and fabrication of printed constructs [73,82,110,111]. A network suitable for printing is therefore not necessarily suitable for long-term culture; effective bioink design requires simultaneous optimization of the processing and culture/degradation timescales.

### 8.5. Flexible Sensors and Actuators

In flexible sensors and actuators, hydration provides tissue-like compliance and a continuous medium for ionic transport, whereas microphase domains and dynamic junctions help preserve mechanical integrity during large deformation. Interfacial adhesion can further stabilize coupling between the device and its substrate or biological interface. This combination is illustrated by WPU-reinforced ionically conductive hydrogels used for adhesive sensing [60] and Fe^3+^–polyphenol PU networks used for near-infrared-triggered actuation [35]. Because device response depends simultaneously on the aqueous phase and the load-bearing network, sensitivity, operating strain, recovery rate, and long-term water retention should be evaluated together. Water therefore plays a dual role: sufficient hydration facilitates ionic transport and compliance, whereas water loss or changes in hydration state can alter conductive pathways, microphase structure, and mechanical response. Device performance should consequently be evaluated not only by initial sensitivity or maximum operating strain but also by long-term hydration stability and signal retention during cyclic operation.

### 8.6. Oil–Water Separation and Other Non-Biomedical Applications

The hydration–interface–network relationship of PU hydrogels also extends to non-biomedical environments. Porous PU hydrogels can exploit a stable hydration layer to achieve low underwater oil adhesion and, in combination with their porous structure, enable oil–water separation [97]. Studies of dual-crosslinked PU coatings further indicate that practical separation performance is also constrained by the mechanical stability of the coating and its long-term durability under hydrated conditions [112]. Thus, whether the relevant boundary is a biological interface or an oil–water interface, the shared design principle is to regulate the organization of interfacial water through segmented chemistry and network structure, with the resulting hydration layer, microphase structure, and network stability jointly governing macroscopic function.

The two application classes share the physical principle of interfacial-water regulation rather than identical performance criteria: oil–water separation primarily concerns underwater oil adhesion, wetting behavior, and long-term separation stability, whereas biological interfaces additionally involve interactions with proteins, cells, and tissues. Across these applications, the common design objective is not to maximize the number of incorporated functions, but to select the hydration, microphase, and network state required under the intended service conditions. Application-specific performance should therefore be interpreted as an outcome of the same composition–hydration–structure–property framework developed in the preceding sections.

## 9. Challenges, Design Principles, and Future Perspectives

Recent advances in segmented architecture and dynamic networks have enabled PU hydrogels to combine mechanical, self-healing, responsive, and interfacial functions [12,19]. The central challenge is now to establish comparable and predictive structure–hydration–property relationships rather than continue reporting isolated high-performance formulations. Because reported properties depend strongly on hydration state, equilibration history, specimen geometry, and loading protocol, future studies should routinely specify these conditions and distinguish strength, toughness, recovery, and fatigue metrics according to their corresponding test definitions [11].

A second challenge is to move beyond total water uptake toward quantitative resolution of water state and hydration-induced structural evolution. Hydrated PU studies have demonstrated changes in hydrogen bonding, segmental mobility, surface morphology, and bulk relaxation [25,36,47], while water-induced microphase separation in PU hydrogels shows that hydration can also be deliberately used to construct new load-bearing morphologies [18]. Establishing causal links among hydration, molecular interactions, microphase evolution, and mechanics will require coordinated wet-state and time-resolved measurements acquired from the same PU hydrogel along a common hydration trajectory, combining spectroscopy, water-state analysis, microscopy or scattering, and mechanical testing [13,25,113]. Such experiments should distinguish simple swelling, pre-existing-domain reconstruction, and genuinely newly developed phase separation. Importantly, the complete progression from changes in water state and competitive hydrogen bonding, through segmental migration and bulk microphase evolution, to the macroscopic response has not yet been resolved quantitatively within a single equilibrium PU hydrogel system. Part of the current mechanistic picture derives from segmented PU, moisture-responsive PUU, or moisture-aged TPU-based materials rather than equilibrium hydrogels [25,51,114]. These systems provide valuable mechanistic analogues but should not be treated as quantitative validation of hydrogel behavior. A priority is therefore synchronized characterization of hydration, molecular interactions, microphase morphology, and mechanics within the same PU hydrogel and over the same temporal window.

A third challenge concerns durability under realistic wet-state service conditions. High water content, strength, toughness, rapid healing, low hysteresis, transport, and dimensional stability are frequently competing requirements. Short-term recovery after a single cut or several unnotched loading cycles does not establish long-term durability; creep, stress relaxation, prolonged immersion, cyclic crack growth, and microphase aging must also be considered [11,25]. The relevant design target is consequently a service-state window in which load-bearing domains and reversible structures remain stable and recoverable over the required timescale. Translation to practical or biomedical use imposes additional constraints as PU-hydrogel network architectures become increasingly complex. Although multiple dynamic interactions and functional additives can improve laboratory performance, they may also complicate synthetic reproducibility, sterilization, batch consistency, processing, and degradation-product control. These issues are particularly important for biomedical PU materials [21,22,98,100]. Sustainability should be considered in parallel with performance and manufacturability. Bio-based PU chemistry and non-isocyanate polyurethane routes offer complementary strategies for reducing dependence on conventional fossil- and isocyanate-based synthesis [100,115], but emerging chemistries must retain the controllable hydrogen bonding, phase heterogeneity, hydration response, and processability required for hydrogel function.

Ultimately, progress toward predictive design will require integration across molecular, mesoscale, and continuum descriptions. Molecular simulations can interrogate competitive polymer–water interactions; mesoscale approaches can address hydration-driven domain evolution; and fracture or transport models can connect evolving network structure with macroscopic function. When combined with standardized experimental datasets, data-driven methods may help map composition and hydration conditions onto structural and property spaces. The central objective is therefore not prediction of a universally optimal PU hydrogel, but identification of molecular and network architectures that generate defined hydrated structural states and maintain the required combination of properties under specified service conditions.

## 10. Conclusions

PU hydrogels are heterogeneous hydrated networks whose properties emerge from the coupling among segmented molecular architecture, hydrophilic–hydrophobic heterogeneity, microphase organization, reversible interactions, and network topology. Soft segments regulate water affinity and molecular mobility, whereas hard-segment-rich domains provide physical crosslinking and load transfer. Hydration is therefore not merely a consequence of swelling but an active structural variable capable of redistributing intermolecular interactions, altering segmental dynamics, and reconstructing microphase organization. The central structure–property pathway identified in this review begins with molecular and network architecture, which determines water state and polymer–water interactions. These hydration-dependent interactions regulate competitive hydrogen bonding and segmental mobility, which in turn govern microphase reconstruction and dynamic-network evolution and ultimately control macroscopic properties and functions. The outcome of hydration is system-dependent: it may promote plasticization and relaxation or, when appropriate structural heterogeneity and network constraints are present, facilitate structural reorganization and reinforcement. Rational PU-hydrogel design should therefore focus on identifying application-specific hydration–network states in which load-bearing integrity, dissipation, recovery, transport, and interfacial behavior remain mutually compatible over the required service timescale. Establishing such relationships through standardized wet-state testing and coordinated in situ multiscale characterization will be essential for moving from empirical formulation optimization toward predictive design.

## Figures and Tables

**Table 1:** Multiscale characterization methods, representative crosslinking and hydration structures, and evidence boundaries for hydration-mediated structural evolution in polyurethane hydrogels.

Mechanistic Aspect	Recommended Characterization	Representative Crosslinking/Network Structure	Representative Hydration Structure/Hydrated-State Feature	Key Observable and Interpretation Boundary	Representative Refs./Level *
Water uptake and water-state heterogeneity	Gravimetric swelling; DSC; dielectric spectroscopy; ^1^H NMR	Covalently crosslinked PU/NIPU networks; phase-separated PEG–POSS networks with hydrophobic domains acting as physical junctions	Heterogeneous hydration involving strongly associated/non-freezing water, freezable bound water, and bulk-like water; preferential hydration of PEG- or ionic-site-rich regions	Water content, freezing behavior, and mobility. Water-state assignments are method-dependent and cannot be inferred from total uptake alone.	[15,16,39,40,41]; A/B
Polymer–water interactions and segmental mobility	Hydrated-state FTIR/Raman; DSC/*T*_g_; dielectric spectroscopy; solid-state NMR	Segmented PU/PUU networks containing hydrogen-bonded hard domains and polar soft-segment regions	Polymer–water H-bonding competes with pre-existing polymer–polymer H-bonds, increasing soft-phase/interfacial mobility and altering local hard-domain stability	H-bond redistribution and mobility changes. Spectral or dynamical changes alone do not establish bulk microphase reconstruction or uniquely identify its molecular origin.	[25,27,28,40,42,43,49,51]; A/B
Near-surface microphase reconstruction	In-liquid AFM; AFM nanomechanics	Segmented PUU or PEG-containing PU with physically associated hard-segment-rich microdomains	Preferential interfacial hydration accompanied by segmental redistribution and reconstruction of near-surface microphase morphology	Hydration-dependent domain morphology and local mechanics. Near-surface rearrangement cannot be directly extrapolated to bulk structure.	[36,44,45,47,48]; B
Bulk microphase evolution and ordering	Wet/in situ SAXS/WAXS; XRD; complementary spectroscopy	Segmented PU networks with hard-domain physical crosslinks; phase-separated or heterogeneous PU networks	Hydration-dependent changes in domain spacing, ordering, crystallinity, and soft/hard-segment redistribution within the bulk	Domain spacing, ordering and crystallinity. Scattering changes may also reflect swelling and do not alone prove new phase separation or assign domain chemistry.	[13,18,25,46,52]; A/B
Water-induced microphase separation	Time-resolved scattering + spectroscopy + morphological/3D analysis	Hydrophilic PEG-PU/hydrophobic PCL-PU network stabilized by multiple H-bonds and bicontinuous phase connectivity	Selective hydration drives in situ formation of newly segregated and interconnected PEG-rich/PCL-rich domains, producing a bicontinuous hydrated morphology	Formation of new segregated domains or connectivity during hydration. Simple swelling or rearrangement of pre-existing domains is insufficient.	[18,46]; A/B
Network relaxation and reversible reconstruction	Stress relaxation; oscillatory/step-strain rheology; cyclic recovery; healing tests	Dynamic covalent and hybrid PU networks containing disulfide or acylhydrazone bonds together with reversible H-bonded junctions	Hydrated mobile regions facilitate reversible bond exchange, segmental rearrangement, and junction reassociation	Relaxation, hysteresis and recovery kinetics. Network dynamics alone do not identify the exchanging interaction or establish hydration as the cause.	[25,31,32,53,54]; A/B
Mechanical consequences and fatigue	Tensile/compressive tests; loading–unloading; fracture and precracked fatigue tests	Bicontinuous H-bonded, dual-crosslinked, or dynamic PU networks containing persistent load-bearing and reversible dissipative junctions	Coexistence of hydrated mobile regions with persistent load-bearing domains enables deformation and energy dissipation while retaining network connectivity	Modulus, toughness, fracture energy and fatigue threshold. Toughness, fracture energy, cyclic recovery and fatigue resistance are not interchangeable metrics.	[11,18,31,55]; A/C
Cross-scale causal resolution	Synchronized time-resolved spectroscopy, NMR/dielectric analysis, scattering and mechanics	System-specific PU hydrogel network characterized consistently across molecular, microphase, and macroscopic scales	Time-resolved coupling of water state, H-bond redistribution, segmental mobility, microphase evolution, and network response	Temporal coupling among hydration, H-bond redistribution, mobility, microphase evolution and macroscopic response. The complete sequence remains unverified within a single equilibrium PU hydrogel system.	[18,25]; A/B, partial

^*^ Evidence levels: A, direct evidence from PU/PUU hydrogels or closely related hydrated crosslinked PU networks; B, PU-specific mechanistic evidence from hydrated segmented PU, PUU, or TPU systems; C, general hydrogel-physics or methodological evidence. Evidence level reflects directness to the proposed hydration mechanism, not study quality. Abbreviations: AFM, atomic force microscopy; DSC, differential scanning calorimetry; FTIR, Fourier-transform infrared spectroscopy; NMR, nuclear magnetic resonance; SAXS/WAXS, small-/wide-angle X-ray scattering; XRD, X-ray diffraction. Note: Ex situ SEM/TEM after dehydration, drying, freezing, or staining should be regarded as supportive morphology rather than direct evidence of hydrated microphase reconstruction unless validated independently by wet/in situ characterization. Representative crosslinking and hydration structures are summarized from the systems included in the corresponding references; hydration states are reported only where supported by the cited experimental evidence.

**Table 2:** Representative quantitative physical and mechanical properties of polyurethane hydrogels under hydrated conditions.

Representative PU Hydrogel	Hydration State/Swelling Metric	Modulus	Strength	Elongation at Break	Toughness, Fracture, and Cyclic Performance
PEG–POSS multiblock PU hydrogel [16]	Equilibrium swelling ratio ≈ 70–600%, depending on PEG/POSS composition	Shear modulus, G ≈ 0.3–4.0 MPa	—	—	—
Triol-crosslinked biodegradable PU hydrogel [29]	Water uptake ≈ 60%	Modulus ≈ 0.9–1.7 MPa	Strength > 3 MPa	>700%	—
PU/TA dual-crosslinked hydrogel (TA30–PU) [55]	Equilibrium-swollen state after immersion in water for 72 h	Elastic modulus ≈ 0.24 MPa	Strength ≈ 2.2 MPa	≈750%	Fracture energy ≈ 9.6 kJ·m^−2^; cyclic recovery efficiency up to ≈96% at 300% strain and ≈87% at 600% strain
Disulfide-containing PEG-based PU hydrogel (PUG-II) [32]	Solvent-exchanged hydrated state; pH-responsive equilibrium swelling	—	Strength ≈ 0.448 MPa; compressive stress ≈ 0.371 MPa at 90% strain	≈489%	After 5 tensile cycles at 200% strain: stress retention ≈ 92.7% and residual strain ≈ 15.9%; 5 compression cycles at 90% strain showed nearly overlapping stress–strain curves
PEG-PU/PCL-PU bicontinuous hydrogel [18]	Water-swollen state after in situ water-induced microphase separation	—	Break strength ≈ 12.9 MPa	—	Toughness ≈ 48.2 MJ·m^−3^; fracture energy ≈ 2.435 kJ·m^−2^

**Note:** Values are reported as provided in the cited studies under hydrated or equilibrium-swollen conditions. Because modulus definitions, specimen geometries, strain rates, hydration conditions, and testing protocols differ among studies, the values are intended for representative comparison rather than direct performance ranking. “—” indicates that the corresponding parameter was not reported or was not directly comparable in the cited study. Cyclic loading–unloading or recovery data are reported as cyclic-performance indicators and should not be interpreted as quantitative fatigue thresholds unless crack-growth-based fatigue testing was performed.

## Data Availability

No new data were created or analyzed in this study. Data sharing is not applicable to this article.
